# Supplementary material for: Infusing theory into deep learning for interpretable reactivity prediction
Source: Nat Commun. 2021 Sep 6;12:5288. doi: 10.1038/s41467-021-25639-8 (PMC8421337; doi:10.1038/s41467-021-25639-8)
Supplement: Supplementary file 1 — Supplementary Information [file 41467_2021_25639_MOESM1_ESM.pdf]

Supplementary Information

Infusing Theory into Deep Learning for Interpretable Reactivity Prediction

Shih-Han Wang†, Hemanth Somarajan Pillai†, Siwen Wang, Luke E. K. Achenie, and Hongliang Xin*

Department of Chemical Engineering,

Virginia Polytechnic Institute and State University, Blacksburg, VA 24061, USA

* hxin@vt.edu

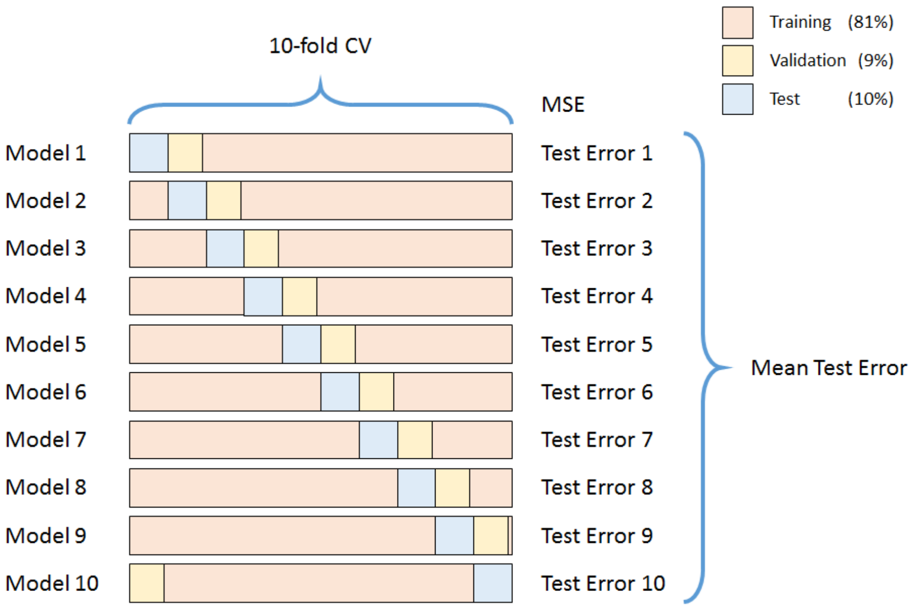

Supplementary Figure 1. Hyper-parameter optimization with the regular 10-fold cross-validation approach.

Supplementary Table I. Hyper-parameters of the ML models optimized by the Ray Tune package.

	FCNN	CGCNN	TinNet
lr	$1.0016\text{x}10^{-3}$	$1.9359\text{x}10^{-3}$	$2.0243\text{x}10^{-3}$
n_conv	-	3	5
n_h	4	4	2
$atom\_fea\_len$	-	55	95
h_fea_len	107	37	174

Supplementary Table II. Average wall-time taken to train a model on Nvidia Tesla P100.

	FCNN	CGCNN	TinNet
Wall-time (sec)	101.34	1,036.87	2,418.72

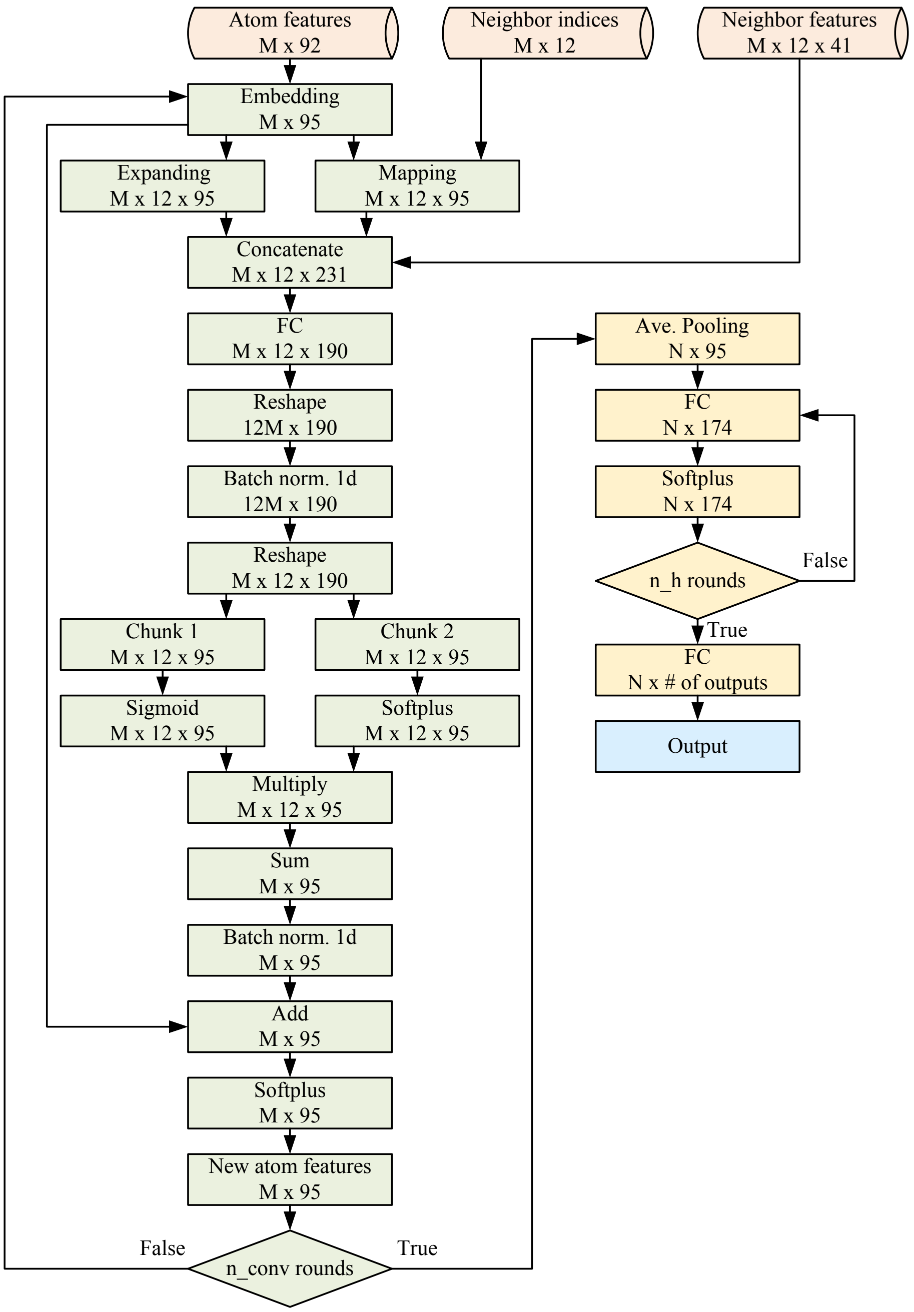

Supplementary Figure 2. TinNet model architecture and hyper-parameters for *OH.

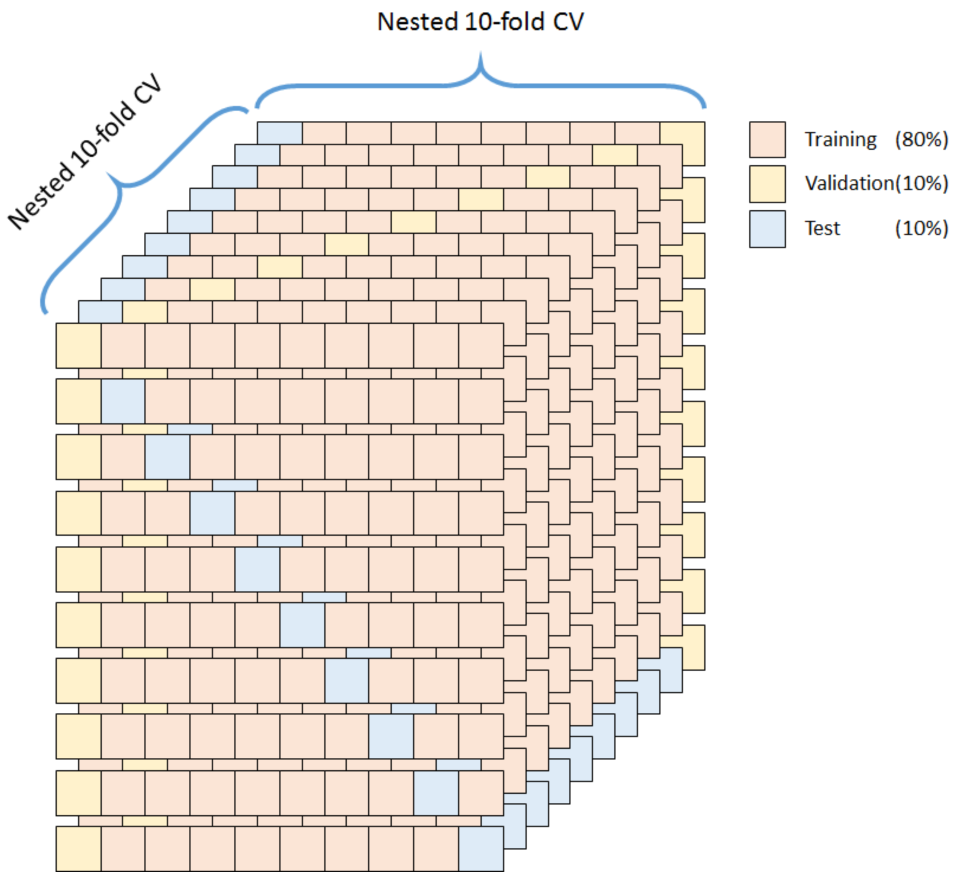

Supplementary Figure 3. Evaluating model performance with the nested 10-fold cross-validation approach. 10 final models were taken when the validation and test folds are the same.

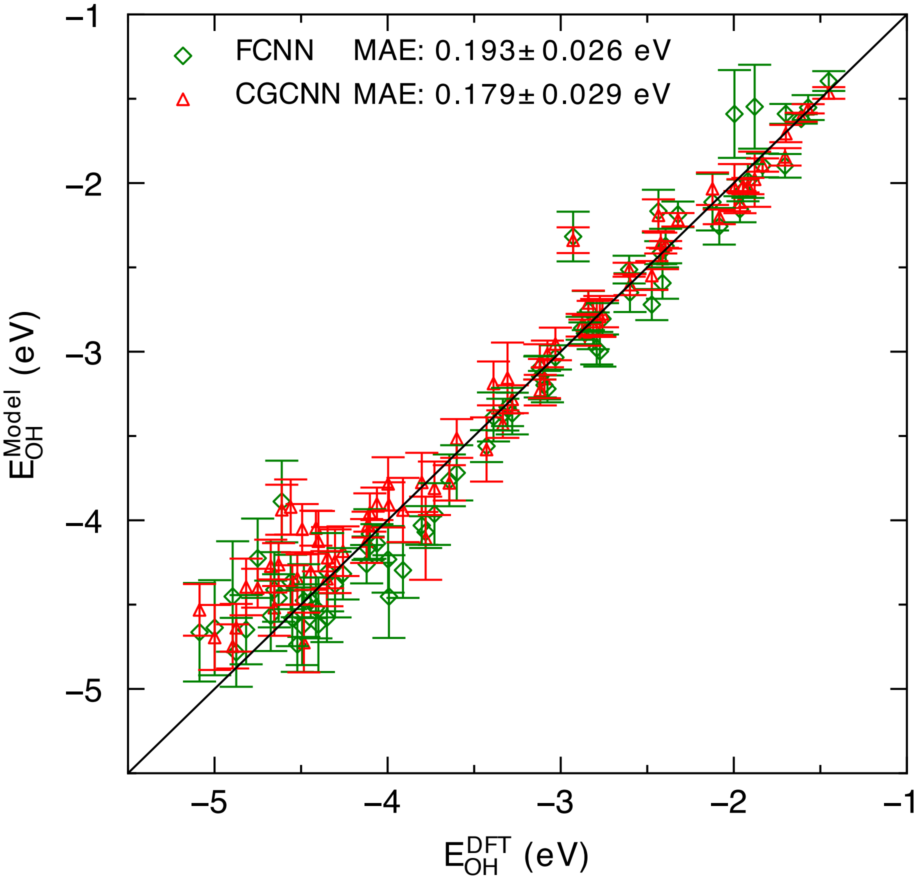

Supplementary Figure 4. DFT-calculated vs. CGCNN- and FCNN-predicted *OH adsorption energies for out-of-sample single-atom alloys. A broad range of transition-metal atoms (26 in total) were used as the single-site substitute of the coinage metal host, i.e., Cu, Ag, and Au.

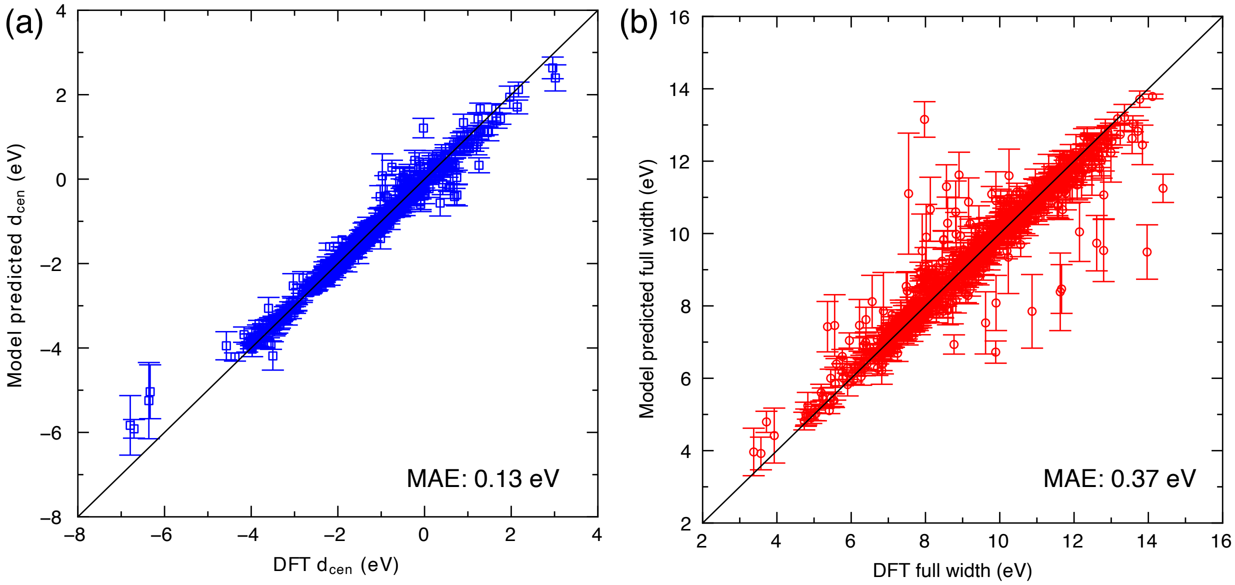

Supplementary Figure 5. Parity plot of DFT-calculated vs. TinNet-predicted (a) d-band center (ε_d) and (b) full-width (W_d) for $\{111\}$-terminated bimetallic surfaces.

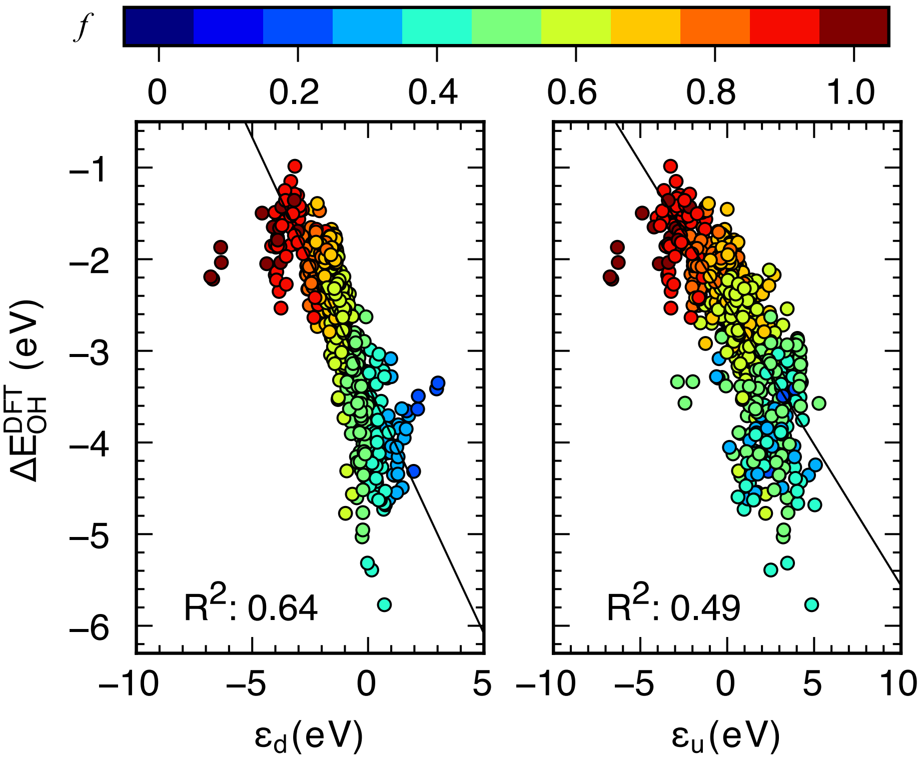

Supplementary Figure 6. DFT-calculated *OH adsorption energies as a function of (a) d-band center (ε_d) and (b) d-band upper edge (ε_u) for all 10-fold test test. The markers are color coded according to the d-band filling f of the *OH adsorption site.

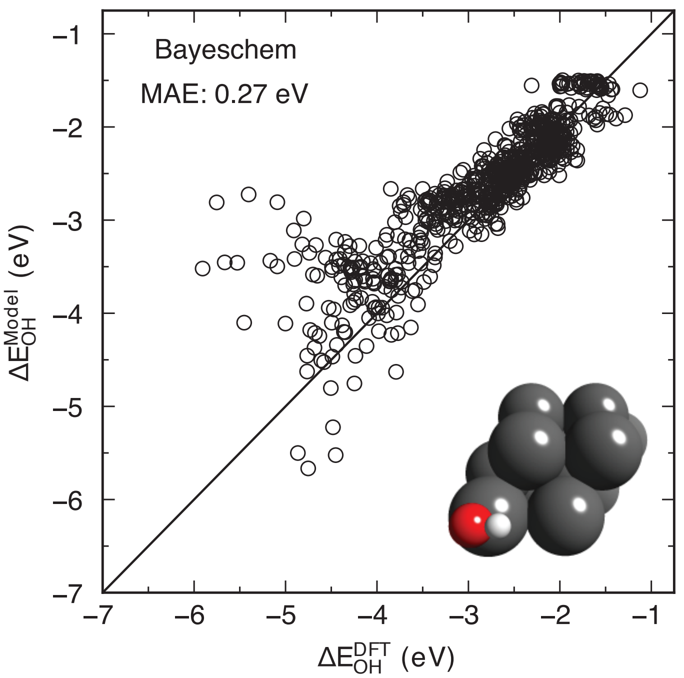

Supplementary Figure 7. Bayschem predicted *OH adsorption energies vs. DFT-calculations for 748 {111}-terminated bimetallic surfaces used in this study.
